# Supplementary figures and images for: Rose (Rosa gallica) Petal Extract Suppress Proliferation, Migration, and Invasion of Human Lung Adenocarcinoma A549 Cells through via the EGFR Signaling Pathway
Source: Molecules. 2020 Nov 4;25(21):5119. doi: 10.3390/molecules25215119 (PMC7663240; doi:10.3390/molecules25215119)

(A)

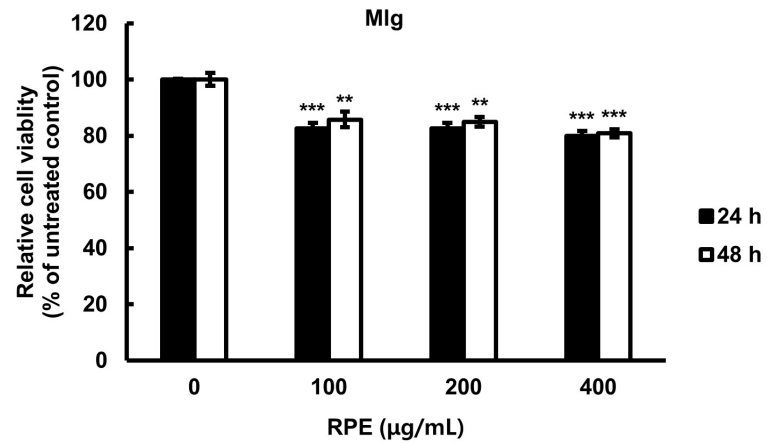

(B)

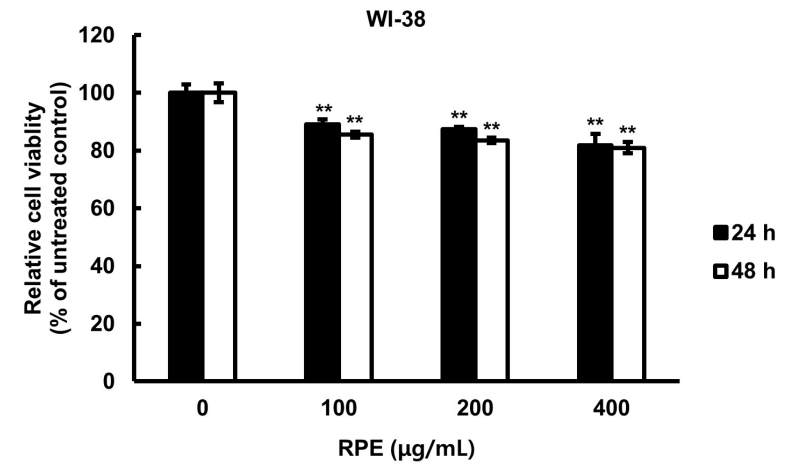

(C)

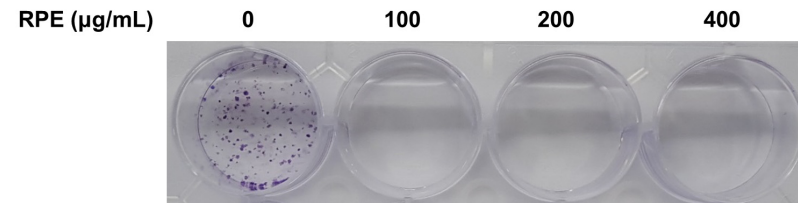

(D)

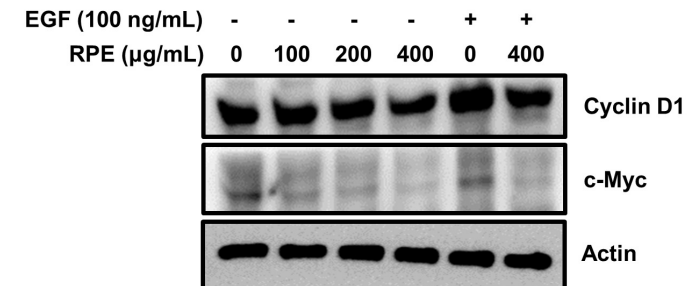

(E)

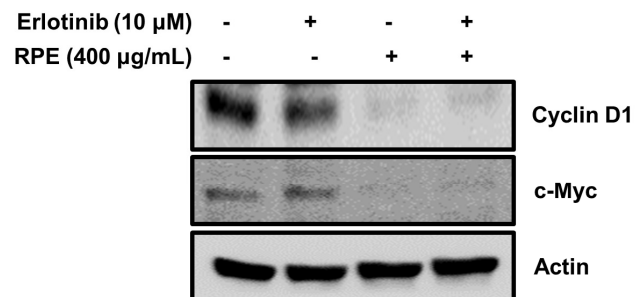

Supplement: Supplementary file 1 [file molecules-25-05119-s001.pdf]
